# Sequential combinations of chemotherapeutic agents with BH3 mimetics to treat rhabdomyosarcoma and avoid resistance

**DOI:** 10.1038/s41419-020-02887-y

**Published:** 2020-08-15

**Authors:** Clara Alcon, Albert Manzano-Muñoz, Estela Prada, Jaume Mora, Aroa Soriano, Gabriela Guillén, Soledad Gallego, Josep Roma, Josep Samitier, Alberto Villanueva, Joan Montero

**Affiliations:** 1 Institute for Bioengineering of Catalonia (IBEC), Barcelona Institute of Science and Technology (BIST), 08028 Barcelona, Spain; 2 Developmental Tumor Biology Laboratory, Institut de Recerca Sant Joan de Déu, 08950 Esplugues de Llobregat, Spain; 3 Department of Haematology and Oncology, Hospital Sant Joan de Déu Barcelona, 08950 Esplugues de Llobregat, Spain; 4 Group of Translational Research in Child and Adolescent Cancer, Vall d’Hebron Research Institute (VHIR), Universitat Autònoma de Barcelona (UAB), 08035 Barcelona, Spain; 5 Department of Surgery, Universitat Autònoma de Barcelona (UAB), 08193 Barcelona, Spain; 6 Department of Electronics and Biomedical Engineering, University of Barcelona (UB), 08028 Barcelona, Spain; 7 Networking Biomedical Research Center in Bioengineering, Biomaterials and Nanomedicine (CIBER-BBN), 28029 Madrid, Spain; 8 Program against Cancer Therapeutic Resistance (ProCURE), IDIBELL, Catalan Institute of Oncology, l’Hospitalet del Llobregat, 08907 Barcelona, Spain; 9 Xenopat S.L., Business Bioincubator, Bellvitge Health Science Campus, l’Hospitalet de Llobregat, 08907 Barcelona, Spain

**Keywords:** Paediatric cancer, Predictive markers

## Abstract

Rhabdomyosarcoma (RMS) is the most common soft tissue sarcoma in childhood and adolescence. Refractory/relapsed RMS patients present a bad prognosis that combined with the lack of specific biomarkers impairs the development of new therapies. Here, we utilize dynamic BH3 profiling (DBP), a functional predictive biomarker that measures net changes in mitochondrial apoptotic signaling, to identify anti-apoptotic adaptations upon treatment. We employ this information to guide the use of BH3 mimetics to specifically inhibit BCL-2 pro-survival proteins, defeat resistance and avoid relapse. Indeed, we found that BH3 mimetics that selectively target anti-apoptotic BCL-xL and MCL-1, synergistically enhance the effect of clinically used chemotherapeutic agents vincristine and doxorubicin in RMS cells. We validated this strategy in vivo using a RMS patient-derived xenograft model and observed a reduction in tumor growth with a tendency to stabilization with the sequential combination of vincristine and the MCL-1 inhibitor S63845. We identified the molecular mechanism by which RMS cells acquire resistance to vincristine: an enhanced binding of BID and BAK to MCL-1 after drug exposure, which is suppressed by subsequently adding S63845. Our findings validate the use of DBP as a functional assay to predict treatment effectiveness in RMS and provide a rationale for combining BH3 mimetics with chemotherapeutic agents to avoid tumor resistance, improve treatment efficiency, and decrease undesired secondary effects.

## Introduction

Rhabdomyosarcoma (RMS) is a highly malignant cancer that, despite being relatively rare, is the most frequent soft-tissue sarcoma in children, accounting for 5% of all pediatric tumors^1^. RMS tumors are highly aggressive and typically develop from skeletal muscle cells, arising in a variety of anatomic sites in the body^2,3^. There is a slightly higher prevalence of this disease in males than in females, and it is often associated with genetic disorders, such as Li–Fraumeni familiar cancer syndrome and neurofibromatosis type 1^2^. Based on histologic criteria, RMS tumors are subdivided into two main groups, embryonal (ERMS) and alveolar (ARMS). ERMS account for 60% of all RMS, affecting children under the age of 10, especially around the head and neck regions^2,3^. ARMS represent ~20% of all RMS, occurring mostly in adolescents and frequently localized in the limbs^3,4^. The current treatment strategies for RMS include chemotherapy, radiation, and surgery^4^. Despite treatment improvement for patients with low-risk and intermediate-risk disease, the survival rates for high-risk patients have not advanced in the past decades^4^. Furthermore, the derived toxicities from current treatments and the lack of biomarkers^5^, highlight the need for new therapies to enhance RMS clinical outcomes.

Therapy causes the death of cancer cells mostly by apoptosis, a process controlled by the BCL-2 family of proteins^6^. Its members are classified based on their structure, BCL-2 homology (BH) domains, and their function^6,7^. In brief, the anti-apoptotic proteins (BCL-2, BCL-xL MCL-1, and others) present up to four BH domains (BH1–BH4) and bind to pro-apoptotic proteins. The pro-apoptotic effector proteins BAX and BAK also contain several BH domains and have the capacity to oligomerize and form pores in the mitochondrial outer membrane. Their function is induced by activator proteins possessing a unique BH3 domain, such as BIM, BID (mostly through the truncated form, tBID), or PUMA. There is a fourth group of BCL-2 family proteins - the so-called sensitizers - also presenting a unique BH3 domain that cannot directly activate effectors, but can inhibit anti-apoptotic members. Sensitizers include BAD, HRK, BIK, NOXA, and BMF - among others - and exert a pro-apoptotic effect by competing for specific binding to anti-apoptotic BCL-2 family proteins^7^. Overall, these proteins regulate mitochondrial outer membrane permeabilization (MOMP) and the release of cytochrome c (and other proteins) that represents the point of no return for apoptotic cell death. Importantly, MOMP can be prevented by anti-apoptotic proteins through direct binding to BAX and BAK or activator BH3-only proteins^7^.

Evasion of apoptosis is a hallmark of human cancers and can be often explained by the increased expression of anti-apoptotic proteins^8,9^. In fact, high levels of BCL-2 and MCL-1 have been reported in RMS patients as a pro-survival mechanism^10,11^. Therefore, targeting anti-apoptotic proteins represents a promising therapeutic approach to treat high-risk or relapsed RMS patients^9,12^. In this regard, BH3 mimetics - a novel class of therapeutics that mimic the action of sensitizer BH3-only proteins and selectively inhibit anti-apoptotic BCL-2 family members^7^ - could be used to overcome apoptotic resistance. There is an increasing interest in BH3 mimetics due to their therapeutic potential alone or in combination with other treatments, but the main questions that clinicians must face are when and how to use BH3 mimetics as anti-cancer therapies in the clinic^7^. On this subject, the functional assay dynamic BH3 profiling (DBP) can determine in <24 h how effective a treatment will be to engage apoptosis^13^. This technology uses synthetic BH3 peptides derived from BCL-2 family proteins to measure how close cells are to the apoptotic threshold (or how primed for death). DBP has been successfully used to predict - from days to weeks in advance - treatment effectiveness in cell lines, murine models, and patient samples^13–17^. In addition to overall susceptibility to apoptosis, DBP can identify the selective dependence of cancer cells on anti-apoptotic proteins, and guide the use of BH3 mimetics to overcome therapy-induced resistance^7^.

Several publications by Fulda and colleagues elegantly demonstrate BH3 mimetics’ therapeutic potential to treat RMS^12,18–20^, although sequential combination of anti-cancer agents with BH3 mimetics has not been fully assessed. Here, we report a new strategy that utilizes low-dose combinations of chemotherapeutic agents with BH3 mimetics to increase the efficacy of current treatments, while decreasing therapy-induced toxicity^21^ and anti-apoptotic protection.

## Materials and methods

### Cell lines and treatments

RMS cell lines (CW9019, RD, and RH4) were kindly provided by Dr. Oscar Martínez-Tirado and Dr. Cristina Muñoz-Pinedo from the Bellvitge Biomedical Research Institute (IDIBELL). C2C12 cells were purchased at ATCC (ATCC^®^ CRL-1772^™^, ATCC, Manassas, VI, USA). Human skeletal muscle myoblasts (HSMM) were purchased at Lonza (CC-2580, Lonza, Basel, Switzerland). RMS cell lines were cultured in RPMI 1640 medium (31870, Thermo Fisher, Gibco, Paisley, Scotland) supplemented with 10% heat-inactivated fetal bovine serum (10270, Thermo Fisher, Gibco), 1% of l-glutamine (25030, Thermo Fisher, Gibco), and 1% of penicillin and streptomycin (15140, Thermo Fisher, Gibco). C2C12 cells were cultured in DMEM high glucose medium (41965, Thermo Fisher, Gibco) supplemented with 10% heat-inactivated fetal bovine serum (10270, Thermo Fisher, Gibco) and 1% of penicillin and streptomycin (15140, Thermo Fisher, Gibco). HSMM cells were cultured in SKBM-2 medium (CC-3246, Lonza) supplemented with its specific SingleQuots^TM^ and growth factors (CC-3244, Lonza). All cells were tested for mycoplasma and maintained at 37 °C in a humidified atmosphere of 5% CO_2_. Drug treatments were performed directly in the culture media at the doses and time points indicated in every single experiment. All drugs were purchased at Selleckchem (Munich, Germany).

### Dynamic BH3 profiling

DBP experiments were performed as previously described^22,23^. In brief, 3 × 10^4^ cells/well in a 96-well plate were used for cell lines. 25 μL of BIM BH3 peptide (final concentration of 0.01, 0.03, 0.1, 0.3, 1, 3, and 10 μM), 25 μL of BAD BH3 peptide (final concentration of 10 μM), 25 μL of HRK BH3 peptide (final concentration of 100 μM), and 25 μL of MS1 BH3 peptide^24^ (final concentration of 10 μM) in MEB (150 mM mannitol, 10 mM hepes–KOH pH 7.5, 150 mM KCl, 1 mM EGTA, 1 mM EDTA, 0.1% BSA, 5 mM succinate) with 0.002% digitonin were deposited per well in a 96-well plate (3795, Corning, Madrid, Spain). Single cell suspensions were stained with the viability marker Zombie Violet (423113, BioLegend, Koblenz, Germany) and then washed with PBS and resuspended in MEB in a final volume of 25 μL. Cell suspensions were incubated with the peptides for 1 h following fixation with formaldehyde and staining with cytochrome c antibody (Alexa Fluor^®^ 647 anti-Cytochrome c—6H2.B4, 612310, BioLegend). Individual DBP analyses were performed using triplicates for DMSO, alamethecin (BML-A150-0005, Enzo Life Sciences, Lorrach, Germany), the different BIM BH3 concentrations used, BAD, HRK, and MS1 BH3 peptides. The expressed values stand for the average of three different readings performed with a high-throughput flow cytometry SONY instrument (SONY SA3800, Surrey, UK). % priming stands for the % of cytochrome c release obtained from different BH3 peptides, and Δ% priming represents the difference between treated cells minus non-treated cells for a given peptide.

### Cell death analysis

Cells were stained with fluorescent conjugates of Annexin V (FITC Annexin V, 640906 or Alexa Fluor^®^ 647 Annexin V, 640912, BioLegend) and propidium iodide (PI) (1056, BioVision, Milpitas, CA, USA) or DAPI (62248, Thermo Fisher) and analyzed on a flow cytometry Gallios instrument (Beckman Coulter, Nyon, Switzerland). Viable cells are Annexin V negative and PI or DAPI negative, and cell death is expressed as 100%-viable cells.

### Protein extraction and quantification

Proteins were extracted by lysing the cells for 30 min at 4 °C using RIPA buffer (150 mM NaCl, 5 mM EDTA, 50 mM Tris–HCl pH = 8, 1% Triton X-100, 0.1% SDS, EDTA-free Protease Inhibitor Cocktail (4693159001 Roche, Mannkin, Germany)) followed by a centrifugation at 16,100 × *g* for 10 min. The supernatant was stored at −20 °C for protein quantification performed using Pierce^TM^ BCA Protein Assay Kit (23227, Thermo Fisher).

### Immunoprecipitation

Cells were lysed in Immunoprecipitation buffer (150 mM NaCl, 10 mM Hepes, 2 mM EDTA, 1% Triton, 1.5 mM MgCl_2_, 10% glycerol, and EDTA-free Protease Inhibitor Cocktail (4693159001 Roche)) and centrifuged at 14,000 × *g*, 15 min at 4 °C. The resulting supernatants were incubated with magnetic beads (161-4021, Bio-Rad, Madrid, Spain) conjugated to 5 µg of rabbit anti-MCL-1 antibody (CST94296, Cell Signaling, Leiden, The Netherlands) or 5 μg of rabbit IgG antibody (CST2729, Cell Signaling) at 4 °C overnight. A fraction of the supernatant (30 μL) was removed and mixed with half volume of 4× SDS–PAGE sample buffer, heated at 96 °C for 5 min and stored at −80 °C as cell lysate fractions. After magnetization, a part of the supernatant was mixed with half volume of 4× SDS–PAGE sample buffer, heated at 96 °C for 5 min and stored at −80 °C as unbound fractions. The rest of the supernatant was discarded. The resulting pellet was washed and mixed with 40 µL 4× SDS–PAGE sample buffer and heated for 10 min at 70 °C to allow the dissociation between the purified target proteins and the beads–antibody complex. The sample was magnetized and the supernatant was collected and stored at −80 °C as IP fractions for further immunoblotting (Western blot) analyses.

### Immunoblotting

Proteins were separated by SDS–PAGE (Mini-Protean TGX Precast Gel 12%, 456-1045, Bio-Rad) and transferred to PVDF membranes (10600023, Amersham Hybond, Pittsburgh, PA, USA). Membranes were blocked with dry milk dissolved in Tris buffer saline with 1% Tween 20 (TBST) for 1 h and probed overnight at 4 °C with the primary antibodies of interest directed against: rabbit anti-BCL-2 (CST4223, Cell Signaling), rabbit anti-BCL-xL (CST2764, Cell Signaling), rabbit anti-MCL-1 (CST5453, Cell Signaling), rabbit anti-BIM (CST2933, Cell Signaling), rabbit anti-BID (CST2002, Cell Signaling), rabbit anti-BAK (CST12105, Cell Signaling), rabbit anti-BAX (CST2772, Cell Signaling), rabbit anti-Actin (CST4970, Cell Signaling) followed by anti-rabbit IgG HRP-linked secondary antibody (CST7074, Cell Signaling) in 3% BSA in TBST for 1 h at room temperature. Immunoblots were developed using Clarity ECL Western substrate (1705060, Bio-Rad). When necessary, immunoblots were stripped in 0.1 M glycine pH 2.5, 2% SDS for 40 min and washed in TBS. Bands were visualized with LAS4000 imager (GE Healthcare Bio-Sciences AB, Uppsala, Sweden) and ImageJ was then used to measure the integrated optical density of bands.

### Development of RMS orthoxenograft mouse model

Six-week-old male athymic nu/nu mice (Envigo, Barcelona, Spain) weighing 18–22 g were used in this study. Animals were housed in a sterile environment, in cages with autoclaved bedding, food, and water. Mice were maintained on a daily 12 h light, 12 h dark cycle. The Institutional Ethics Committees approved the study protocol, and the animal experimental design was approved by the IDIBELL animal facility committee (AAALAC Unit1155). All experiments were performed in accordance with the guideline for Ethical Conduct in the Care and Use of Animals as stated in The International Guiding Principles for Biomedical Research Involving Animals, developed by the Council for International Organizations of Medical Sciences.

An embryonal rhabdomyosarcoma (ERMS) orthoxenograft was generated from a small biopsy of a metastatic case taken at diagnostic from the primary tumor located in the child gluteus. The patient gave written consent to participate in the study. The primary tumor did not receive radiotherapy or chemotherapy prior to surgery. Under isoflurane anesthesia, a subcutaneous pocket was made with surgical scissors. Then, a small incision was made in the muscle and the tumor was fixed with synthetic monofilament, non-absorbable polypropylene suture (Prolene 7.0) to the muscle of the upper thigh (orthotopic implantation). After implantation, tumor formation was checked weekly by palpation. Orthotopic tumor (named RMSX1) became apparent 1–3 months after engraftment. Once orthotopic tumors had reached a volume of around 1500 mm^3^, mice were sacrificed and tumors were passed to another three animals in order to obtain a sufficient quantity of tumor material. After each passage tumors were frozen, paraffin-embedded, and cryopreserved in (10% DMSO + 90% non-inactivated fetal bovine serum (10270, Thermo Fisher, Gibco)) to provide a source of viable tissue for future experiments.

### Drug treatment in ERMS RMSX1 orthoxenograft tumor model

The orthoxenograft procedure was approved by the campus Animal Ethics Committee and complied with Association for Assessment and Accreditation of Laboratory Animal Care International (AAALAC) procedures. A mouse harboring RMSX1 tumor - orthotopically growing, at passage#2 - was sacrificed, tumors were harvested and cut into small fragments 4 × 4 mm^3^, and the tumor fragments were grafted in 20 young mice. When tumors reached a homogeneous size (1200–1500 mm^3^), mice were randomly allocated into the different treatment groups (*n* = 4/group): (i) Placebo; (ii) ABT-199 (100 mg/kg); (iii) vincristine (1 mg/kg); (iv) S63845 (20 mg/kg); and (v) combined vincristine (1 mg/kg) plus S63845 (20 mg/kg). Vincristine was intravenously administrated via tail vein injection (i.v.) once per week for 3 consecutive weeks (days 0, 7, and 14). ABT-199 was daily administered (q.d.) by oral gavage (p.o.) for 21 days and S63845 was i.v. administered 3 consecutive days per week for 2 weeks. All the animals/groups were sacrificed at day 21. To minimize in combined treatments the risk of developing drug-induced toxicity, drugs were administered spaced in time. Vincristine was administered first and S63845 2 h later. Vincristine from Eli Lilly (1 mg/ml) was purchased at the hospital pharmacy of the Catalan Institute of Oncology (ICO) and diluted in saline before use. ABT-199 and S63845 were purchased at Selleckchem. ABT-199 was diluted in 10% Ethanol/30% PEG 400/60% Phosal 50 PG (v/v/v), while S63845 was diluted in 10% DMSO/40% PEG 300/5% Tween 80/saline. After treatment initiation, tumors were measured using a caliper every 2–3 days and tumor volume was calculated using the formula *v* = (*w*² × *l)*/2, where *l* is the longest diameter and *w* the width. At the moment of sacrifice, tumor was dissected out and weighed. Representative fragments were either frozen in nitrogen or fixed and then processed for paraffin embedding.

### PDX cell isolation

Primary tumors from PDX animals were exposed to an enzymatic digestion after mechanical disaggregation in 2.5 mL of DMEM media with 125 units of DNAse I (DN25, Sigma-Aldrich, Buchs, Switzerland), 100 units of Hyaluronidase (H3506, Sigma-Aldrich), and 300 units of collagenase IV (17104-019, Thermo Fisher, Gibco). The tissue suspension was processed using gentleMACS Dissociator (Miltenyl Biotec, Madrid, Spain) using the hTUMOR 1 program. The suspension was incubated at 37 °C for 30 min with constant agitation. Then, the program hTUMOR 1 was ran again and repeated the 30 min incubation. We filtered the suspension with a 70 micron filter into a 50 mL conical and cells were spun down at 500 × *g* for 5 min. To lyse the residual red blood cells, 100 µL of ice cold water was added for 15 s and then diluted to 50 mL with PBS, then cells were spun down again. Cells were finally resuspended in RPMI media, counted by trypan blue exclusion and plated in a 12-well plate, 3 × 10^4^ cells/well and treated with DMSO or vincristine 1 nM. After a 16 h incubation at 37 °C in a humidified atmosphere of 5% CO_2,_ DBP analyses were performed.

### Statistical analysis

Statistical significance of the results was analyzed using Student’s *t*-tail test. *p < 0.05 and **p < 0.01 were considered significant. SEM stands for Standard Error of the Mean. For ROC curve analysis, cell lines were considered responsive to treatment when Δ% cell death > 20%. Drug synergies were established based on the Bliss Independent model as previously described^25^. Combinatorial index (CI) was calculated CI = ((*D*_A_ + *D*_B_) − (*D*_A_*D_B_))/*D*_AB_, where *D* represents cell death of compound A or B or the combination of both. Only the combination of drugs with a CI < 1 were considered synergies. GraphPad Prism8 was used to generate the graphs and to perform the statistical analyses.

## Results

### Novel chemotherapy combinations with BH3 mimetics to increase RMS cytotoxicity

**Fig. 1:**
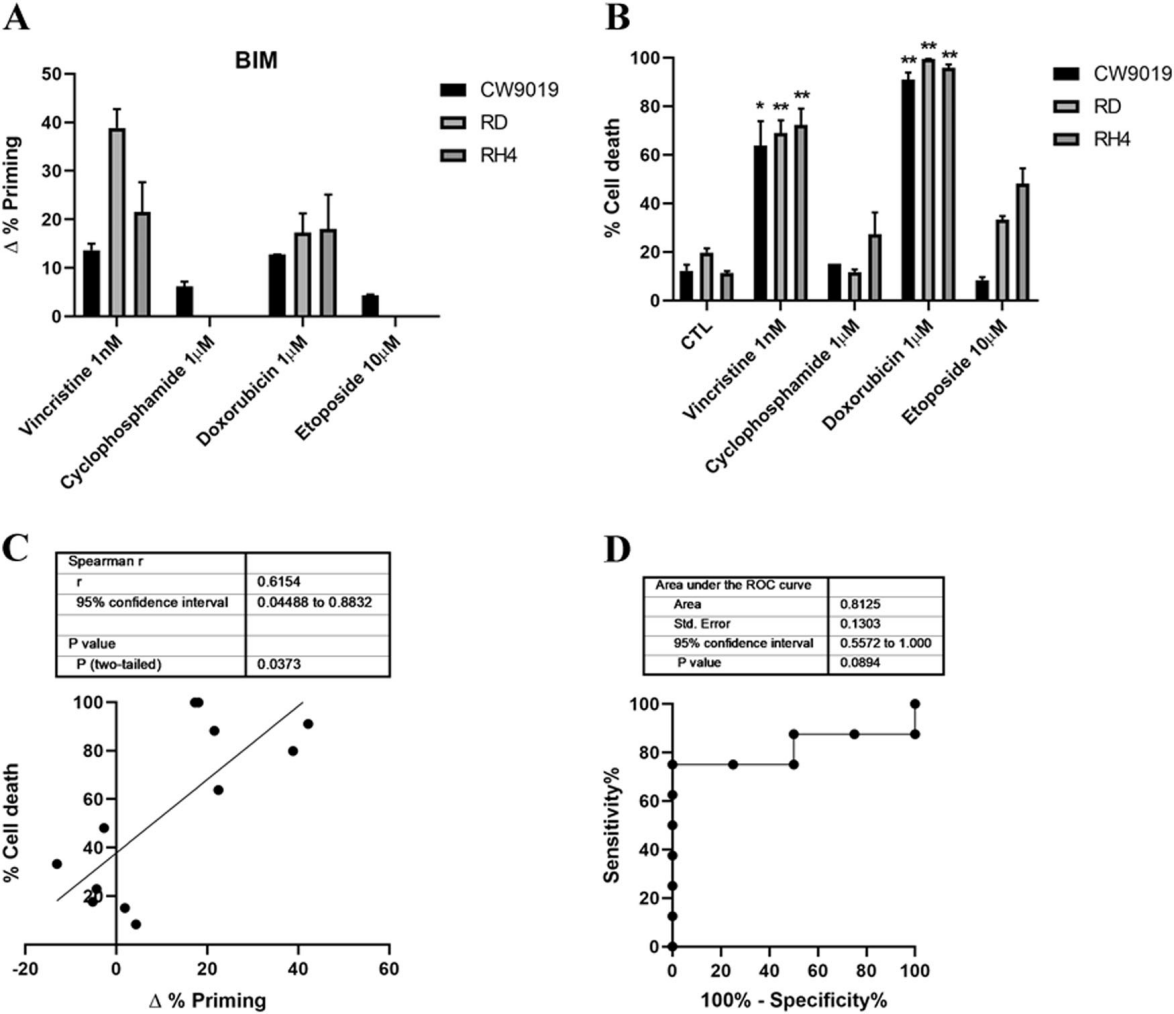
Dynamic BH3 profiling predicts chemotherapy sensitivity in different RMS cell lines. **a** Results from the DBP assay after 36 h incubation with the treatments in CW9019, RD, and RH4 cells. Results expressed as ∆% priming represents the increase in priming compared to control cells. **b** Cell death results from Annexin V and propidium iodide/DAPI staining FACS analyses after 96 h incubation with the chemotherapeutic agents in CW9019, RD, and RH4 cells. **c** Correlation between ∆% priming at 36 h and % cell death at 96 h. **d** Receiver operating characteristic curve analysis. Values indicate mean values ± SEM from at least three independent experiments. **p < 0.01 and *p < 0.05.

Chemotherapeutic agents are commonly used in clinical protocols for RMS treatment^4^. However, they negatively impact patients with short-term and long-term therapy toxicities^26^, and often treatment resistance is acquired by cancer cells^27^. Therefore, we focused on reducing chemotherapeutic dosage to decrease therapy-associated undesired effects. First, we used DBP to analyze the increase in priming after incubation with four standard of care RMS chemotherapeutic agents: the microtubule-destabilizing agent vincristine, the alkylating molecule cyclophosphamide, the anthracycline doxorubicin, and the topoisomerase inhibitor etoposide^28^. We performed DBP on three different RMS cell lines to account for the disease heterogeneity: two ARMS cell lines (CW9019 and RH4) and an ERMS cell line (RD). In CW9019 cells, we observed an increase in apoptotic priming upon treatment (∆% priming) after a short incubation with vincristine and doxorubicin, but not with cyclophosphamide or etoposide (Fig. 1a). Using Annexin V and PI or DAPI staining, we analyzed by flow cytometry cell death after 96 h of exposure to the same chemotherapeutic agents as a proof of principle to evaluate the correlation between DBP predictions and later cell death. We observed high levels of cell death (between 40% and 80%) after vincristine treatment and even nearly complete elimination of cells with doxorubicin, but no effect with cyclophosphamide or etoposide, confirming DBP predictions (Fig. 1b). Similar results were obtained in the other two RMS cell lines, RD and RH4 (Fig. 1a, b). When we statistically compared Δ% priming and % cell death in all three cell lines, we observed a significant correlation (Fig. 1c). To further determine how good DBP is as a binary predictor for RMS, we performed a Receiver Operating Characteristic (ROC) curve analysis^29^. We observed that the area under the curve (AUC) for our experiments was 0.81 (Fig. 1d), indicating that DBP presents a good predictive capacity for chemotherapy cytotoxicity in the RMS cell lines tested.

**Fig. 2:**
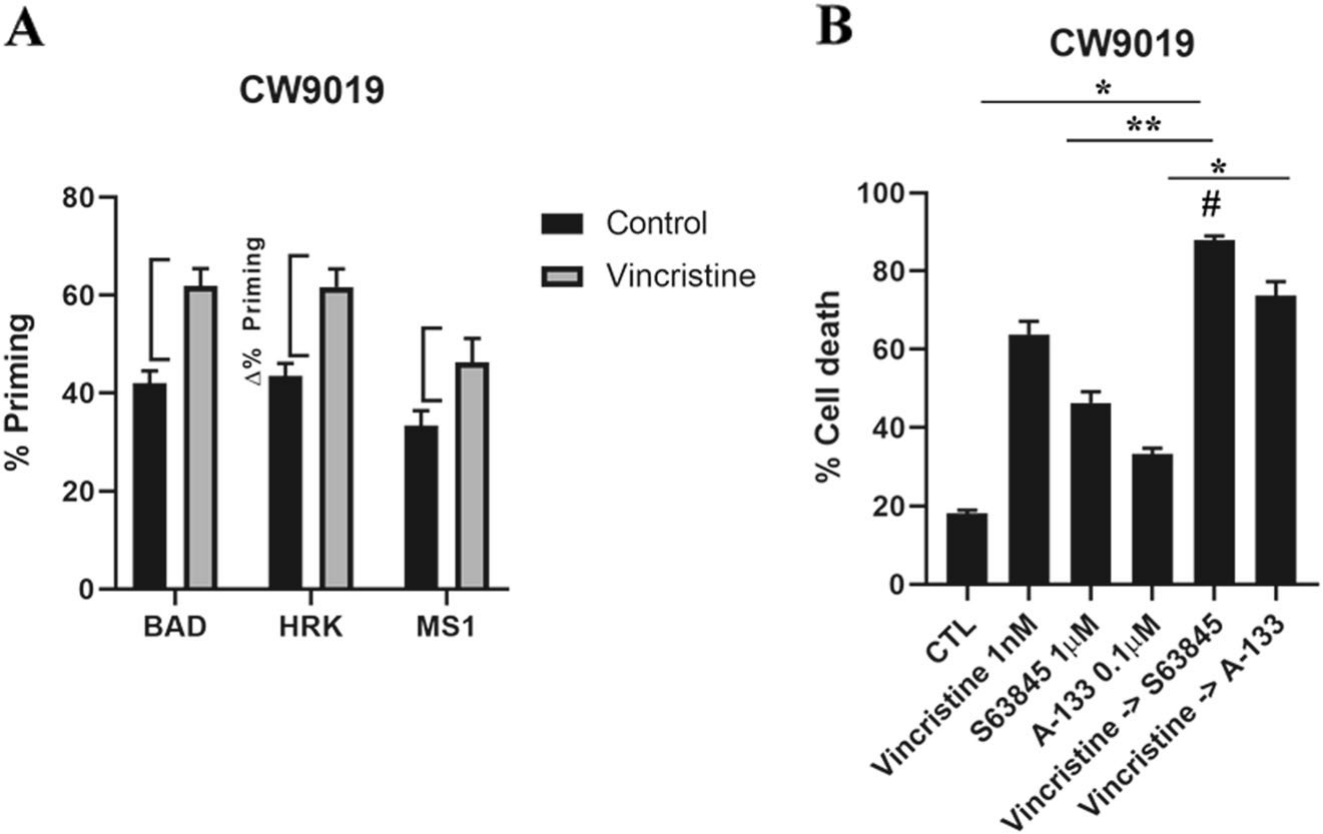
Dynamic BH3 profiling predicts synergistic combinations with vincristine and BH3 mimetics in the CW9019 cell line. **a** Results from the contribution of each anti-apoptotic protein: BCL-2/BCL-xL dependence BAD peptide; BCL-xL dependence HRK peptide; and MCL-1 dependence MS1 peptide in acquiring resistance to vincristine 1 nM treatment. Results expressed as ∆% priming represents the increase in priming compared to control cells. HRK and MS1 BH3 peptides showed a significant increase, indicating BCL-xL and MCL-1 adaptation, respectively. **b** Cell death from Annexin V and propidium iodide staining FACS analyses after 96 h incubation of CW9019 cells with single agents or the combination of vincristine (1 nM) with the corresponding BH3 mimetics S63845 (1 µM) and A-1331852 (0.1 µM) for 96 h. Values indicate mean values ± SEM from at least three independent experiment. **p < 0.01, *p < 0.05 compared to single agents and # indicates CI < 1.

**Fig. 3:**
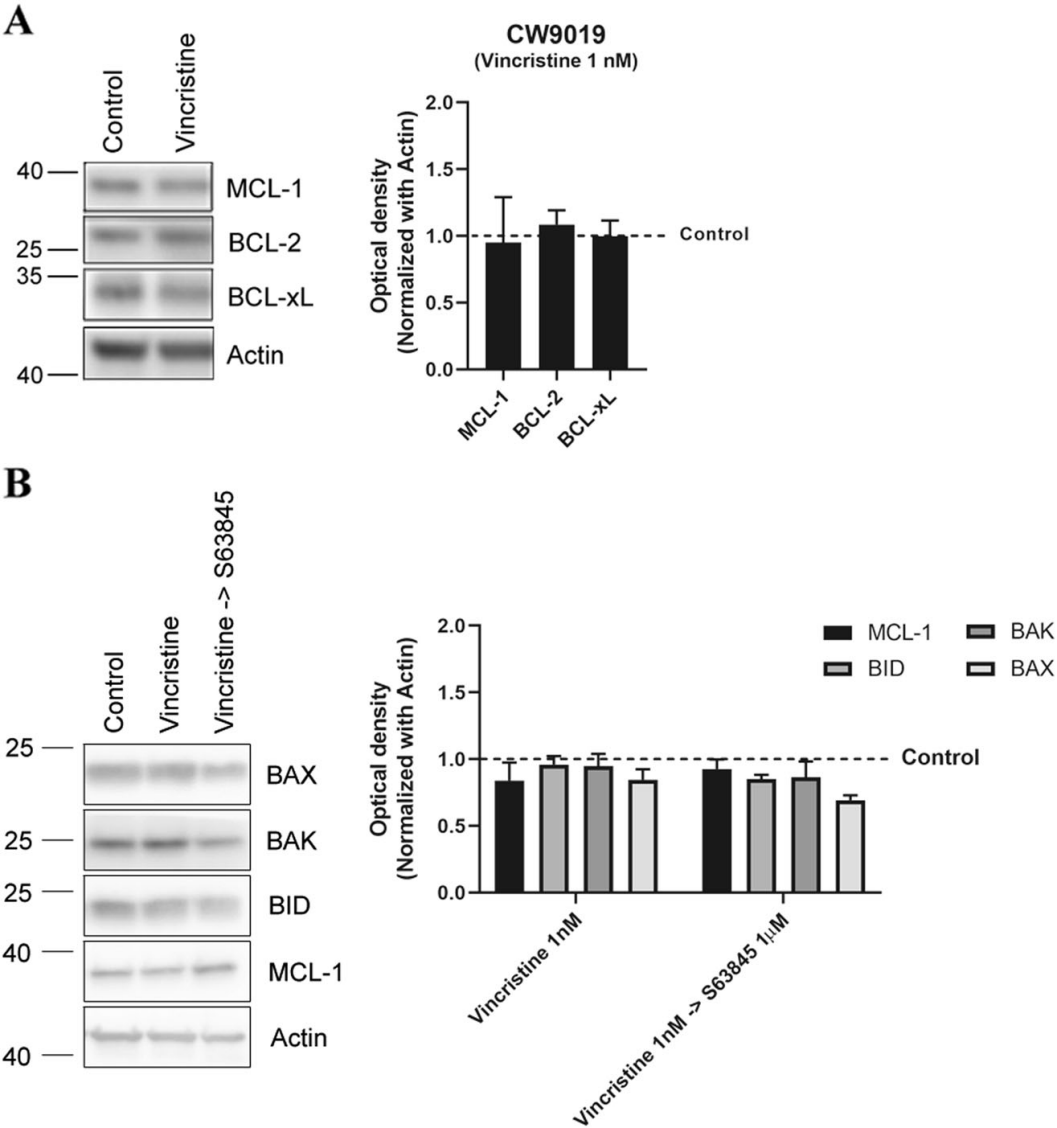
Vincristine treatment subtly affects BCL-2 family proteins. **a** Anti-apoptotic protein levels analysis by Western blot from control CW9019 cells and after the treatment with 1 nM vincristine for 36 h. **b** Effector and activator BCL-2 family protein levels analysis by Western blot from control CW9019 cells, after vincristine (1 nM) and the sequential combination with S63845 (1 µM) for 36 h. Quantification of the optical density of each protein and normalized with actin. Results expressed as fold increase represents the increase in optical density compared to control cells. Values indicate mean values ± SEM from at least three independent experiments.

As mentioned above, one of the hallmarks of cancer is treatment adaptation and resistance to anti-cancer drugs^27^. This resistance can be acquired by different mechanisms such as drug target alterations (mutations), drug export transporters’ gain, increased DNA damage repair, altered proliferation and - as we further investigated - through anti-apoptotic BCL-2 proteins^30^. Using specific synthetic BH3 peptides, that mimic sensitizer BCL-2 family proteins, with DBP we can identify which is the anti-apoptotic protein that cancer cells rely on to acquire resistance to a given treatment^7^. In this regard, we can precisely evaluate the contribution of three main pro-survival BCL-2 family members: BCL-2/BCL-xL dependence with the BAD BH3 peptide, BCL-xL dependence with the HRK BH3 peptide, and MCL-1 dependence with the MS1 BH3 peptide^7,22–24,31^. First, we observed that CW9019 cells present low initial BCL-2, BCL-xL, and MCL-1 dependence (Fig. 2a) based on the basal priming observed in control cells^6^. However, we identified that these cells experimented an increase in % priming, referred as ∆% priming with BAD, HRK, and MS1 BH3 peptides upon vincristine treatment (Fig. 2a), pointing to BCL-xL and MCL-1 mediated pro-survival adaptation. In consequence, we decided to pharmacologically exploit this anti-apoptotic dependence utilizing two new selective BH3 mimetics: S63845 (MCL-1 inhibitor)^32^ and A-1331852 (A-133) (BCL-xL inhibitor)^33^ and test their cytotoxic effect in combination with vincristine. We observed that sequentially adding S63845 or A-1331852 after 36 h of exposure to vincristine significantly increased cell death at 96 h compared to single agents (Fig. 2b). In fact, the combination index (CI) calculations^25^ indicated that S63845 addition to vincristine is synergistic (CI = 0.916) while A-1331852 is additive (CI = 1.028). Obtaining synergy between two agents is an important goal to decrease treatment toxicity and to avoid undesired side effects associated with high doses of chemotherapy, a constant challenge for pediatric cancer^25^. We repeated these experiments with another RMS standard chemotherapeutic agent, doxorubicin, and we observed an increase in ∆% priming with DBP (Fig. 1a) and a high percentage of cell death with Annexin V and DAPI staining (Fig. 1b). Like vincristine, we could detect an increase in priming with BAD, HRK, and MS1 BH3 peptides in CW9019 cells (Supplementary Fig. 1a) indicating that cancer cells also acquired resistance to doxorubicin treatment through BCL-xL and MCL-1. Doxorubicin is already a potent chemotherapeutic drug as a single agent and exerts an extensive cytotoxicity after 96 h (Fig. 1b), but also causes cardiotoxicity in the clinic^21^. Therefore, we sought to reduce doxorubicin dosing by exploring synergistic sequences with the anti-apoptotic inhibitors A-1331852 and S63845. Hereof, doxorubicin combined with both BH3 mimetics was highly cytotoxic at 96 h for RMS cells, even when reducing ten-fold its concentration (Supplementary Fig. 1B). Both combinations of doxorubicin with S63845 or A-1331852 were synergistic as we observed a CI = 0.522 and CI = 0.836, respectively. These combinations were effective in RMS cell lines but we did not observe any cytotoxic effect in non-tumoral myoblast cells (Supplementary Fig. 2), indicating specific toxicity of these treatments for malignant cells. Additionally, we analyzed different anti-apoptotic BCL-2 family proteins expression to determine molecular fluctuations after vincristine and doxorubicin treatments. Surprisingly, we found that upon vincristine treatment there were no significant changes in the anti-apoptotic proteins MCL-1, BCL-xL, or BCL-2 expression, indicating that cancer cells’ adaptation to this therapy relies on different mechanisms other than increased expression of these proteins (Fig. 3a). On the other hand, doxorubicin treatment led to a different adaptation: marked decline in MCL-1 and BCL-xL levels, an increase in BCL-2, BAD, and BIM expression and a decrease in the pro-apoptotic protein BID (Supplementary Fig. 3). From this first set of experiments we conclude that we can increase chemotherapeutic agents’ efficacy by rationally combining them with specific BH3 mimetics. Despite not being the most synergistic combination observed, but taking into account the cardiotoxicity caused by doxorubicin^21^, we postulated the sequential treatment of vincristine and S63845 as the most promising and effective therapy for RMS, and we further studied it (Fig. 2b).

### Vincristine promotes MCL-1-mediated resistance

**Fig. 4:**
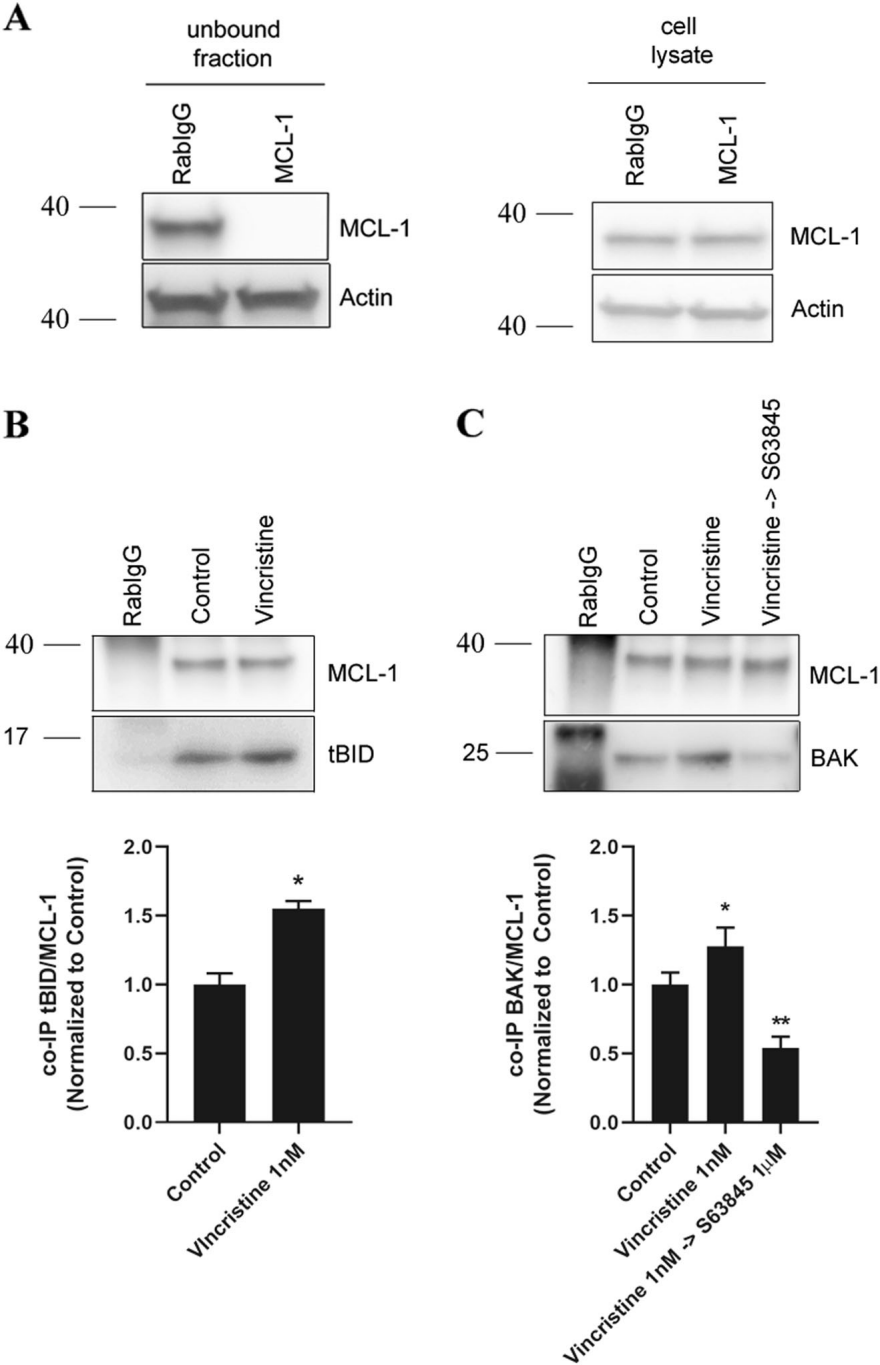
Vincristine induces resistance in RMS cells through BID and BAK inhibition by MCL-1. **a** Left panel: Western blot results of the unbound fraction after MCL-1 immunoprecipitation. Right panel: MCL-1 levels in the initial cell lysates. High efficiency of MCL-1 immunoprecipitation compared to Rabbit IgG control antibody. **b** Western blot results of the co-immunoprecipitation between MCL-1 and tBID in control conditions and after 1 nM vincristine treatment for 36 h. Results showed a significant increase in tBID and MCL-1 binding after vincristine treatment. **c** Western blot results of the co-immunoprecipitation between MCL-1 and BAK in control conditions, after 1 nM vincristine treatment and after the sequential combination of 1 nM vincristine and 1 µM S63845 for 36 h. Results showed a significant increase in BAK and MCL-1 binding after vincristine treatment, which was decreased below control levels after the addition of S63845. Values indicate mean values ± SEM from at least three independent experiments. **p < 0.01, *p < 0.05.

To better understand the molecular adaptation by which cells acquire resistance to vincristine and why its combination with S63845 is highly effective in vitro, we analyzed MCL-1 interactions with other BCL-2 family proteins by performing immunoprecipitation assays. First, we found that there were no significant expression changes in effectors and activator BCL-2 family proteins in cell lysates when exposed to vincristine or its sequential combination with S63845 (Fig. 3b). Using CW9019 cells we observed a significant MCL-1 decrease in the flow through fraction despite similar MCL-1 levels in initial cell lysates (Fig. 4a), and a good detection in the pulled down samples (Fig. 4c, d). We assessed MCL-1 binding with activator proteins BIM and BID after vincristine treatment. As previously described, MCL-1 can bind to the truncated form of BID (tBID), prevent BAX and BAK activation, MOMP, and apoptosis^34^. Indeed, we could observe an increase in binding between MCL-1 and tBID after vincristine treatment (Fig. 4b) but not with BIM (Supplementary Fig. 4A). When we studied BCL-2 family effector proteins we observed that vincristine treatment caused a significant increase in MCL-1 binding with BAK (Fig. 4c) but not with BAX (Supplementary Fig. 4B). These observations correlate with previous reports showing tBID preferential activation of BAK over BAX^35^, and MCL-1 sequestration of BAK as a resistance mechanism to anti-cancer treatments^36,37^. Importantly, this enhanced binding could be reversed by S63845 addition after vincristine treatment (Fig. 4c), decreasing MCL-1:BAK binding below control levels and recovering CW9019 cells’ apoptotic function. Vincristine treatment increases priming and pro-apoptotic proteins tBID and BAK binding to MCL1, which can be released by S63845. These findings demonstrate why the sequential combination of vincristine and S63845 is effective against RMS.

### Effective therapeutic combination in vivo of vincristine with the MCL-1 inhibitor S63845

**Fig. 5:**
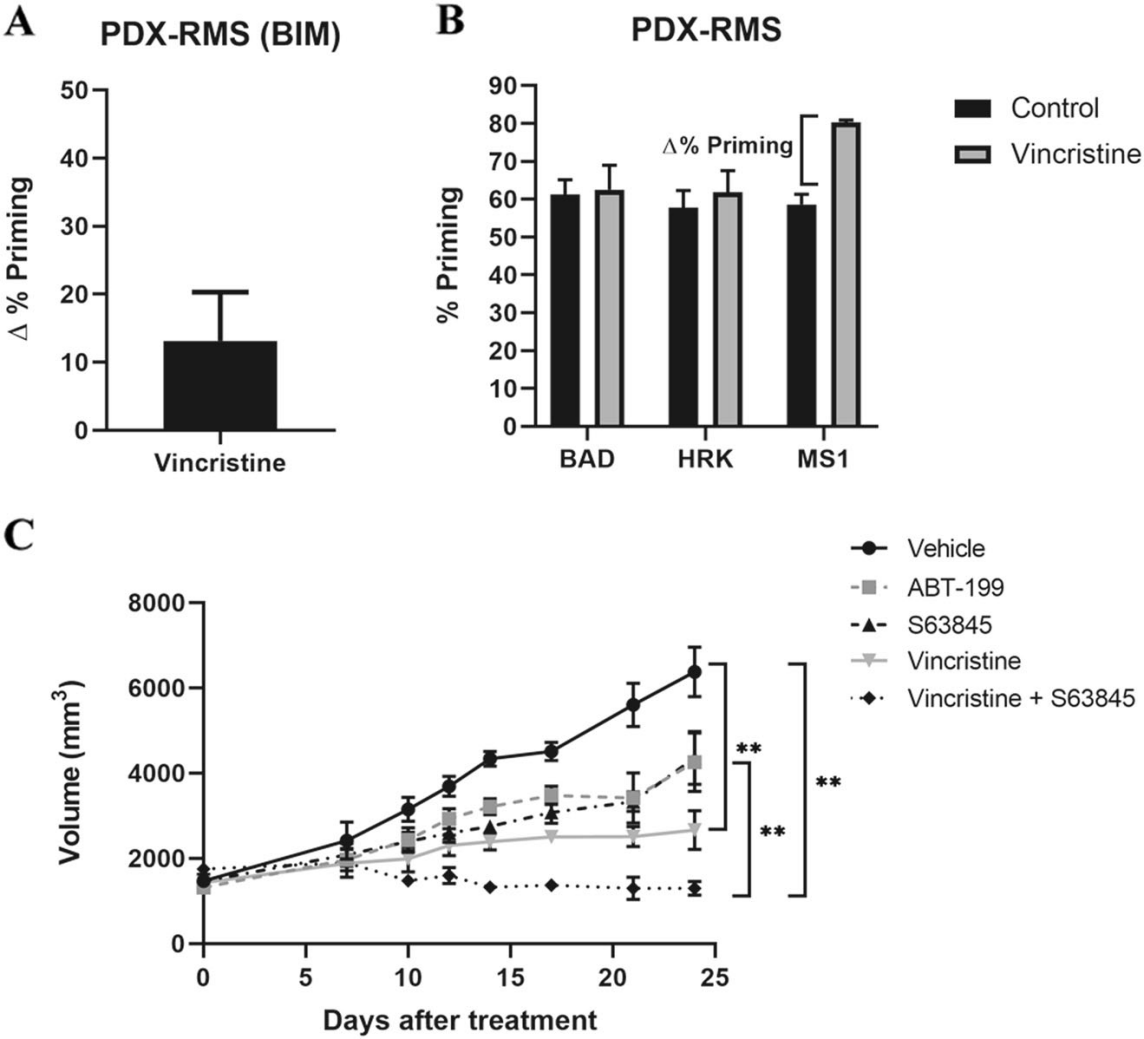
Sequential treatment of vincristine and S63845 stops tumor progression in the PDX model of RMS RMSX1. **a** DBP results of PDX cells from RMS cancer patient showing an increase in ∆% priming after vincristine treatment. Results expressed as ∆% priming represents the increase in priming compared to control cells. *n* = 2. **b** DBP results of PDX cells from a RMS cancer patient with the sensitizer peptides. MS1 BH3 peptide showed a significant increase, indicating MCL-1 adaptation. *n* = 2. **c** Tumor growth results after 21 days of treatment with vehicle, vincristine, the BH3 mimetics S63845 and ABT-199 and the combination of vincristine and S63845. Day 0 indicates the day animals received the treatments. All values indicate mean values ± SEM. **p < 0.01, *n* = 3.

Patient-derived xenografts (PDXs) are advantageous in pre-clinical research as they recapitulate patients’ therapeutic response^26^. After identifying different effective combinations in vitro, we analyzed tumors from RMS PDX models. We disaggregated the tumors to obtain a single-cell suspension and performed DBP analyses to evaluate different therapies’ effectiveness and possible anti-apoptotic adaptations. We focused on chemotherapeutic agents, particularly on vincristine as it is used in the clinic to treat RMS and because we already generated promising preliminary results in vitro in combination with S63845 (Fig. 1). We analyzed an ERMS orthoxenograft generated from a small biopsy from the primary tumor located in the gluteus of a metastatic patient, that we named RMSX1. We detected an increase in ∆% priming after incubating tumor cells with vincristine (Fig. 5a), but not with cyclophosphamide or etoposide (Supplementary Fig. 5A), similarly to what we observed in cell lines (Fig. 1). Moreover, we identified by DBP an anti-apoptotic adaptation to vincristine mediated by MCL-1 (Fig. 5b) that could diminish its efficacy, as previously observed in vitro (Fig. 2). RMSX1 in vivo single agent treatment with vincristine or the MCL-1 inhibitor S63845 merely delayed tumor growth after 21 days (Fig. 5c). Surprisingly, we detected that the sequential combination of vincristine followed by S63845 was significantly more effective than single agents and promoted tumor reduction in vivo (Fig. 5c and Supplementary Fig. 5C). Moreover, we could also observe a significant increase in ∆% priming after incubating tumor cells with targeted agents, such as S63845, ABT-199, and SP2509 (Supplementary Fig. 5A) and we also identified possible anti-apoptotic adaptations to those treatments by BCL-xL and MCL-1 (Supplementary Fig. 5B) that we will further explore. Overall, these results demonstrate DBP’s efficacy to design more effective therapeutic strategies to overcome anti-apoptotic resistance and avoid cancer progression using BH3 mimetics.

## Discussion

There is an urgent medical need to find more effective and less toxic treatments for RMS patients, since recurrent malignancies present poor prognosis and the overall survival after relapse is very low^31^. There is a growing evidence that the BCL-2 family of proteins (particularly the anti-apoptotic members) may mediate drug resistance in cancer cells causing disease progression in patients^7,8^. Therefore, it is key to predict these acquired pro-survival mechanisms and overcome them with anti-apoptotic inhibitors like BH3 mimetics. As mentioned above, DBP, beyond measuring a given treatment effectiveness to engage apoptosis, can also detect anti-apoptotic adaptations derived from therapy that promote cancer survival^13^, and guide the use of BH3 mimetics to avoid resistance. Anti-apoptotic inhibitors such as A-1331852 (BCL-xL selective), ABT-199 (BCL-2 selective), S63845 (MCL-1 selective) among others that are now evaluated in the clinic, can be used as single agents or especially in combination with other therapies to enhance cancer elimination^7^. In particular, highly potent and selective MCL-1 inhibitors such as S64315 (also named MIK665, similar to S63845)^32^, AZD-5991^38^, and AMG-176^39^, are currently explored in clinical trials and hold great promise for cancer treatment. Therefore, in this study we aimed to investigate the use of BH3 mimetics to boost RMS sensitivity to current chemotherapy.

At present, radiotherapy, surgery, and chemotherapy are the standard of care for RMS treatment. Regarding the latter, a three-drug combination is currently utilized: vincristine, actinomycin D, and cyclophosphamide (VAC). This regimen has become the basis for RMS therapy with the incorporation of other agents, such as etoposide, doxorubicin, ifosfamide, cisplatin, and others for intermediate risk patients, with scarce clinical outcome improvement^40^. However, secondary effects derived from chemotherapy administration in children are severe and may include infertility, cardiomyopathy, or the appearance of secondary neoplasia^40^. One explanation for these unbearable therapy-associated pediatric toxicities relies on differential apoptotic priming between young and adult tissues^21^. Traditional chemotherapy has reached an efficacy plateau in RMS, making development of new therapies that increase efficacy while decreasing toxicity a clear unmet need. Thus, we sought to identify possible mechanisms of resistance to chemotherapeutic agents, such as vincristine or doxorubicin that could explain their limited clinical efficacy by analyzing anti-apoptotic changes with DBP (Fig. 2 and Supplementary Fig. 1). Indeed, we identified anti-apoptotic adaptations to common therapies and tested them in combination with BH3 mimetics to achieve a high cytotoxicity, around 80%, while decreasing ten-fold their concentration, thus their potential secondary effects. More precisely, we found novel synergistic combinations of vincristine with the MCL-1 inhibitor S63845, and doxorubicin with the same BH3 mimetic or the BCL-xL inhibitor A-1331852 (Fig. 2 and Supplementary Fig. 1); the effectiveness of this last combination was also observed in osteosarcoma^41^. These three new combinations were synergistic as assessed by CI index (CI < 1) and allowed dosing reduction^40^. Furthermore, all treatments and combinations with BH3 mimetics reported in this work do not cause cell death neither in the non-tumoral cell line C2C12 (Supplementary Fig. 2A) nor in HSMM (Supplementary Fig. 2B), reinforcing the idea of reducing treatment dosage. These results strengthen the importance of BCL-xL and MCL-1 as therapeutic targets in pediatric cancer, and specifically in RMS as it has also been recently reported^42^. The described treatments have been explored in multiple adult cancers^23,43^, but not in pediatric cancers, where current treatments present low effectiveness especially in high risk and relapsed RMS patients^40^. Previous studies in RMS also demonstrated that different BH3 mimetics can potentiate chemotherapeutic treatment effectiveness^18^, when combined with an ATP-competitive mTOR inhibitor^20^ or a histone deacetylase inhibitor^12^, which supports exploring these therapies as new approximations to treat pediatric patients.

**Fig. 6:**
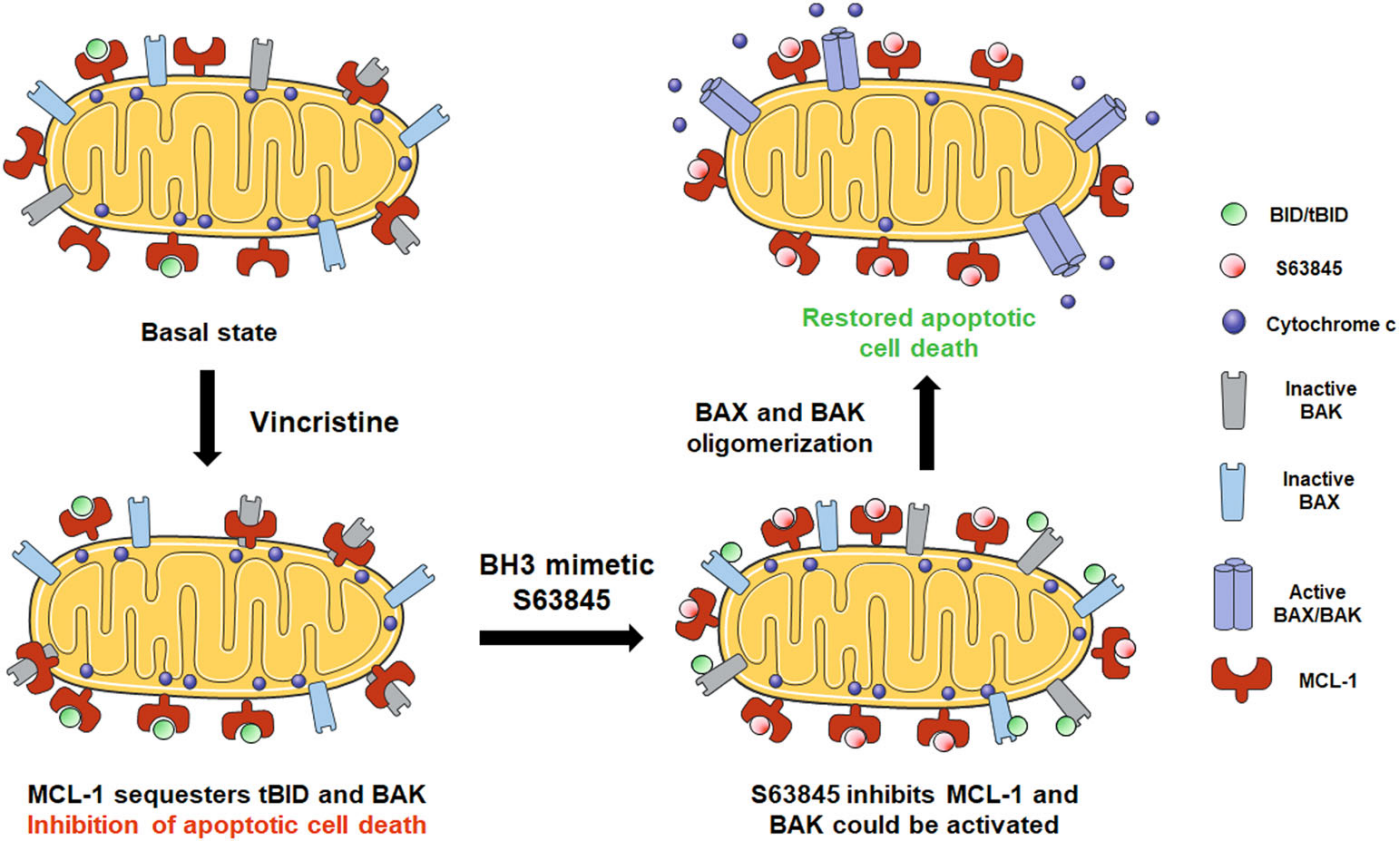
Use of S63845 to overcome RMS cells’ resistance to vincristine. When RMS cells are exposed to vincristine, they adapt to therapy by increasing the binding of BID/tBID and BAK to MCL-1. By blocking MCL-1, using the BH3 mimetics S63845, BAK will be displaced from MCL-1, and BAX and BAK will be able to oligomerize and restore vincristine-induced apoptosis.

As previously mentioned, vincristine is currently used for RMS treatment^40^, but we observed that cells acquire resistance through the anti-apoptotic protein MCL-1. Therefore, we focused our efforts on testing vincristine effectiveness in combination with the MCL-1 inhibitor S63845 in vivo. As PDXs accurately model patients’ outcome^26^, we used a RMS PDX model to test the sequential combination of low dose vincristine therapy with S63845. First, we confirmed using DBP in PDX-isolated cancer cells that vincristine resistance was mediated through MCL-1 (Fig. 5b), correlating with our previous observations in vitro (Fig. 2). When a combination of vincristine followed by S63845 was sequentially administered, these PDXs showed a significant reduction on tumor growth with a tendency to its stabilization (Fig. 5c and Supplementary Fig. 5C), in accordance with the high cytotoxicity observed in vitro (Fig. 2b). To further explain this therapeutic strategy efficacy, we analyzed MCL-1 and NOXA expression but we could not detect significant changes on those proteins (Fig. 3 and data not shown), pointing to another anti-apoptotic mechanism driving the acquired resistance to vincristine. MCL-1 exerts its anti-apoptotic function by sequestering pro-apoptotic BCL-2 family proteins, such as activators BIM/tBID or effectors like BAX/BAK, preventing MOMP and avoiding apoptosis^34–36^. When analyzing RMS cells, we observed a significant increase in MCL-1 binding to tBID by co-immunoprecipitation after vincristine treatment (Fig. 4b). tBID promotes apoptosis by preferentially binding to the effector protein BAK^35^, therefore MCL-1 could decrease treatment effectiveness. But MCL-1 can also bind to BAK to protect cells from apoptosis^36,37^. Interestingly, we could also observe an increase in MCL-1 binding to BAK after vincristine treatment which could be reversed by the sequential addition of S63845 (Fig. 4c). MCL-1 augmented binding to tBID and BAK explains CW9019 acquired resistance to vincristine and why the sequential combination of this chemotherapeutic agent with S63845 restores cytotoxicity both in vitro and in vivo. Thus, our findings demonstrate that MCL-1 inhibition after vincristine treatment is critical to allow MOMP and restore apoptosis in these cells (Fig. 6).

In summary, the work that we here present demonstrates DBP’s capacity to predict days in advance the cytotoxic effect of specific treatments in RMS cells. More interestingly, it can identify how RMS cancer cells acquire resistance to therapy and new approaches to overcome dynamic anti-apoptotic adaptations. To our knowledge, this is the first time that multiple effective sequential combinations of chemotherapeutics with BH3 mimetics are reported for RMS in the same study. The specific capacity of DBP to predict resistance to treatments could be key to personalize patient’s therapy and to avoid toxicities derived from ineffective combinations of treatments that do not promote cancer cell death but undesired side effects. We demonstrated in pre-clinical models (Figs. 2b and 5c) the synergistic antitumor activity of the MCL-1 inhibitor S63845 when sequentially combined with vincristine as was previously identified by DBP (Figs. 2a and 5b). Furthermore, this sequential combination will allow the reduction of chemotherapeutic dosing, which is essential to decrease the secondary effects derived from therapy. These findings, together with the current efforts to target the anti-apoptotic protein MCL-1, currently explored in clinical trials^44^, manifest the importance of rationally combining anti-cancer agents with BH3 mimetics. These novel therapeutic strategies could improve treatment of RMS patients in the clinic, especially for those that relapsed, when guided by a functional predictive biomarker such as DBP.

## Supplementary information

Supplementary Figure legends

Supplementary Figure 1

Supplementary Figure 2

Supplementary Figure 3

Supplementary Figure 4

Supplementary Figure 5
