# Supplementary material for: Sequential combinations of chemotherapeutic agents with BH3 mimetics to treat rhabdomyosarcoma and avoid resistance
Source: Cell Death Dis. 2020 Aug 15;11(8):634. doi: 10.1038/s41419-020-02887-y (PMC7429859; doi:10.1038/s41419-020-02887-y)
Supplement: Supplementary file 1 — Supplementary Figure legends [file 41419_2020_2887_MOESM1_ESM.docx]

**Supplementary figures**

***Supplementary Figure 1: Dynamic BH3 profiling predicts synergistic combinations with doxorubicin and BH3 mimetics in CW9019 cell line.*** *(A) Studying the contribution of each anti-apoptotic protein after 1µM doxorubicin treatment. Results expressed as ∆% priming represent the increase in priming compared to control cells. HRK and MS1 BH3 peptides showed a significant increase in priming, indicating BCL-xL and MCL-1 adaptation respectively. (B) Cell death from Annexin V and DAPI staining and FACS analysis after 96 hours incubation of CW9019 cells with the single agents alone or the combination of doxorubicin (0.1µM) with the BH3 mimetics S63845 (1µM) and A-1331852 (0.1µM). Values indicate mean values ± SEM. ** p< 0.01, * p<0.05 compared to single agents and # indicates CI<1. All experiments were performed at least three times.*

***Supplementary Figure 2: Chemotherapeutics combined with BH3 mimetics have no effect in non-tumoral cells.*** *Cell death from Annexin V and propidium iodide/DAPI staining and FACS analysis after 96 hours incubation with the single agents alone or the combination of vincristine (1nM) or doxorubicin (0.1µM) with the corresponding BH3 mimetics S63845 (1µM) and A-1331852 (0.1µM) for 96 hours in (A) C2C12 cells and (B) Human skeletal muscle myoblasts (HSMM).. Values indicate mean values ± SEM. All experiments were performed at least three times.*

***Supplementary Figure 3: Doxorubicin treatment causes different alterations in BCL-2 family proteins.*** *Left panel: images from Western blot analysis from control CW9019 cells and after the treatment with 1µM doxorubicin for 36 hours. Right panel: Quantification of the optical density of each protein and normalized with actin. Results expressed as fold increase represents the increase in optical density compared to control cells. Values indicate mean values ± SEM. All experiments were performed at least three times.*

***Supplementary Figure 4: Vincristine treatment did not affect binding affinity of MCL-1 with BIM and BAX.*** *Western blot results from MCL-1 immpunoprecipitated samples from control and treated CW9019 lysates. (A) MCL-1 binding to BIM is not affected by 1nM vincristine treatment during 36 hours. (B) Binding between MCL-1 and BAX is very loose in all conditions and is not affected by vincristine treatment. Values indicate mean values ± SEM. All experiments were performed at least three times.*

***Supplementary Figure 5****:* ***Identification of effective treatments as single agents and combinations with BH3 mimetics in a PDX model of RMS.*** *(A) DBP results of PDX cells from RMS cancer patient showing an increase in ∆% priming after S63845, ABT199 and SP2509 but not after cyclophosphamide or etoposide treatment. Results expressed as ∆% priming represents the increase in priming compared to control cells. (B) DBP results of PDX cells from RMS cancer patient with the sensitizer peptides HRK BH3 peptide and MS1 BH3 peptide showed an increase after all treatments, indicating BCL-xL and MCL-1 adaptation. (C) Image of tumors dissected from PDX mice after 25 days of treatment.*
