# Supplementary figures and images for: Sequential combinations of chemotherapeutic agents with BH3 mimetics to treat rhabdomyosarcoma and avoid resistance
Source: Cell Death Dis. 2020 Aug 15;11(8):634. doi: 10.1038/s41419-020-02887-y (PMC7429859; doi:10.1038/s41419-020-02887-y)

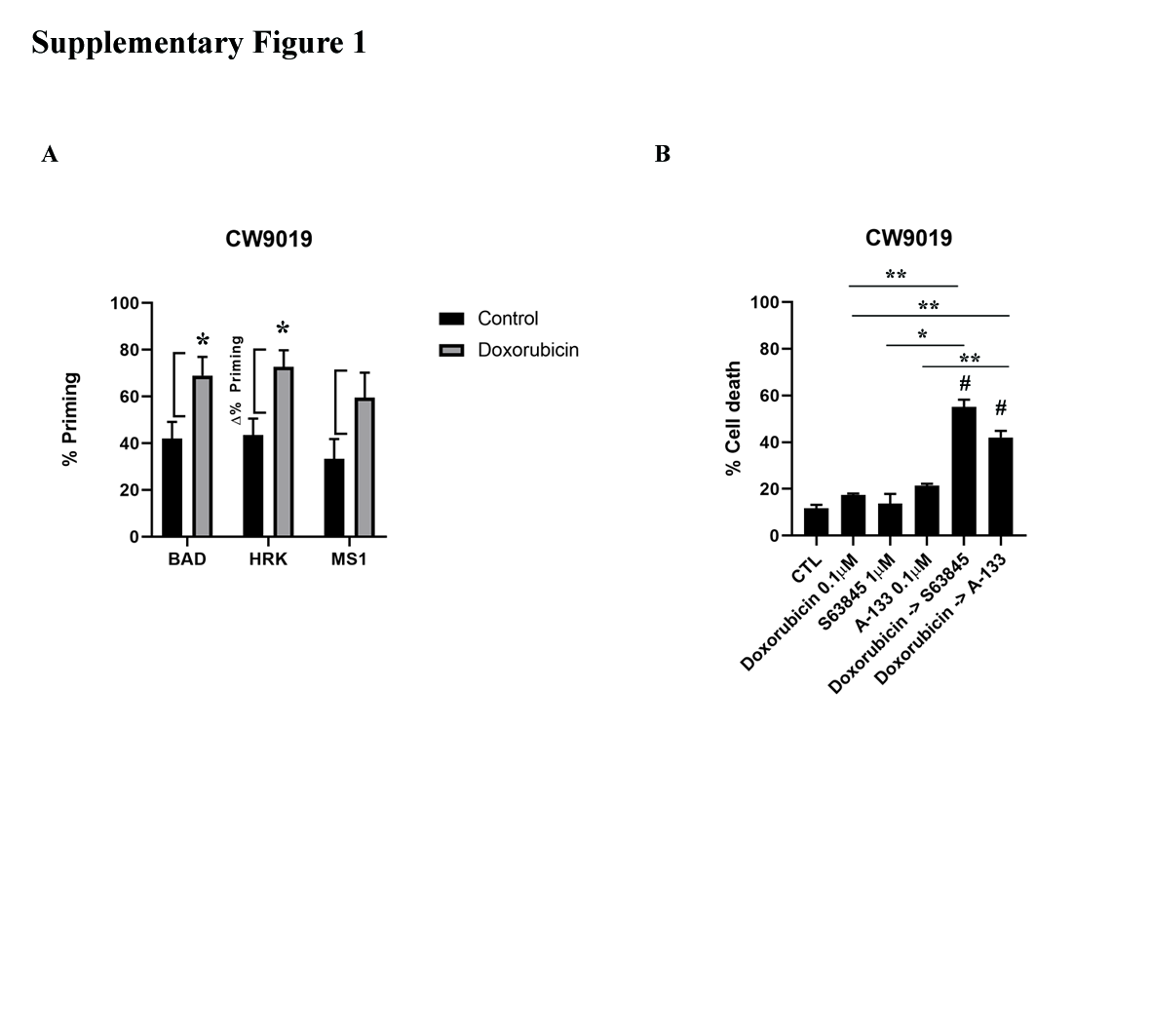

Supplement: Supplementary file 2 — Supplementary Figure 1 [file 41419_2020_2887_MOESM2_ESM.tif]

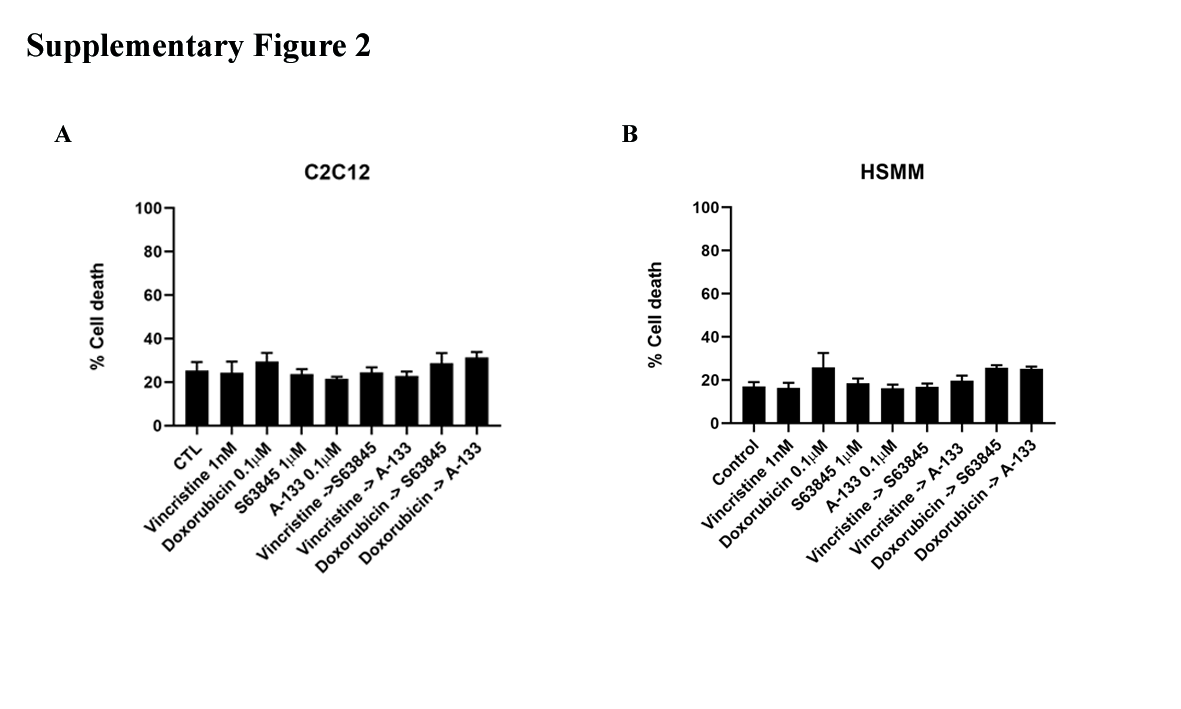

Supplement: Supplementary file 3 — Supplementary Figure 2 [file 41419_2020_2887_MOESM3_ESM.tif]

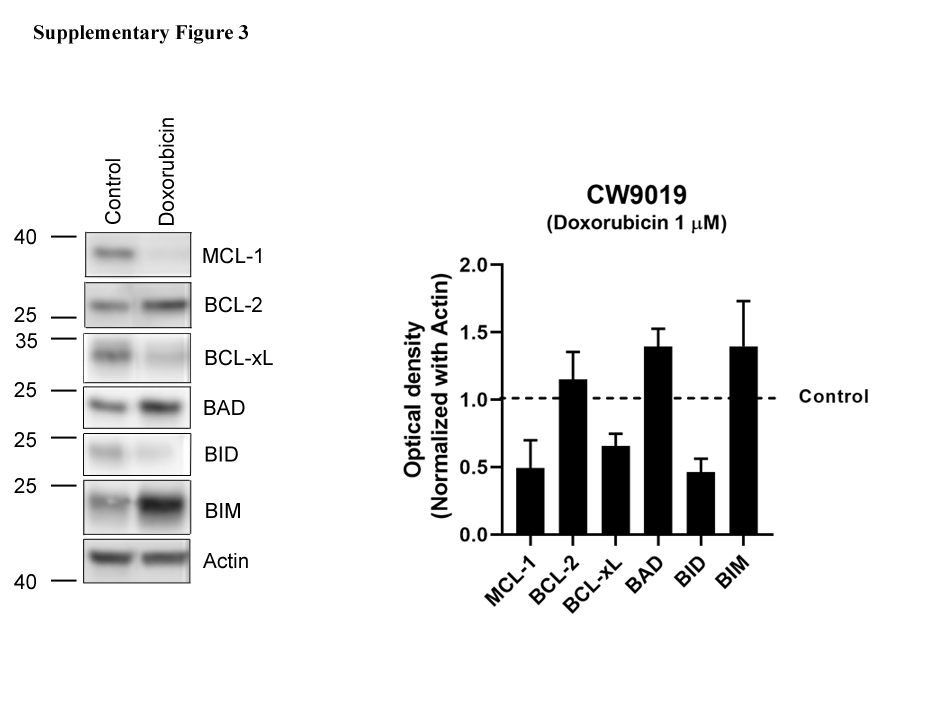

Supplement: Supplementary file 4 — Supplementary Figure 3 [file 41419_2020_2887_MOESM4_ESM.tif]

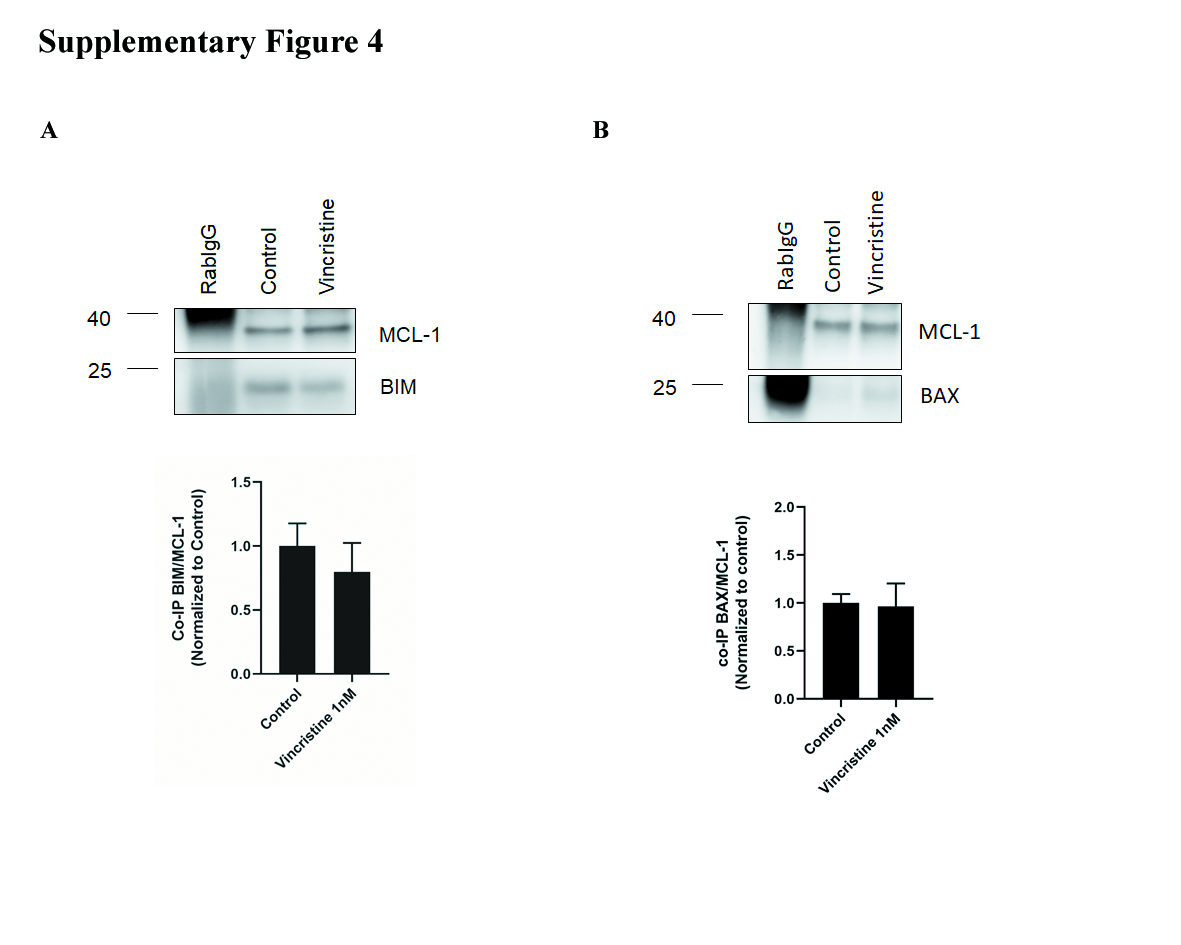

Supplement: Supplementary file 5 — Supplementary Figure 4 [file 41419_2020_2887_MOESM5_ESM.tif]

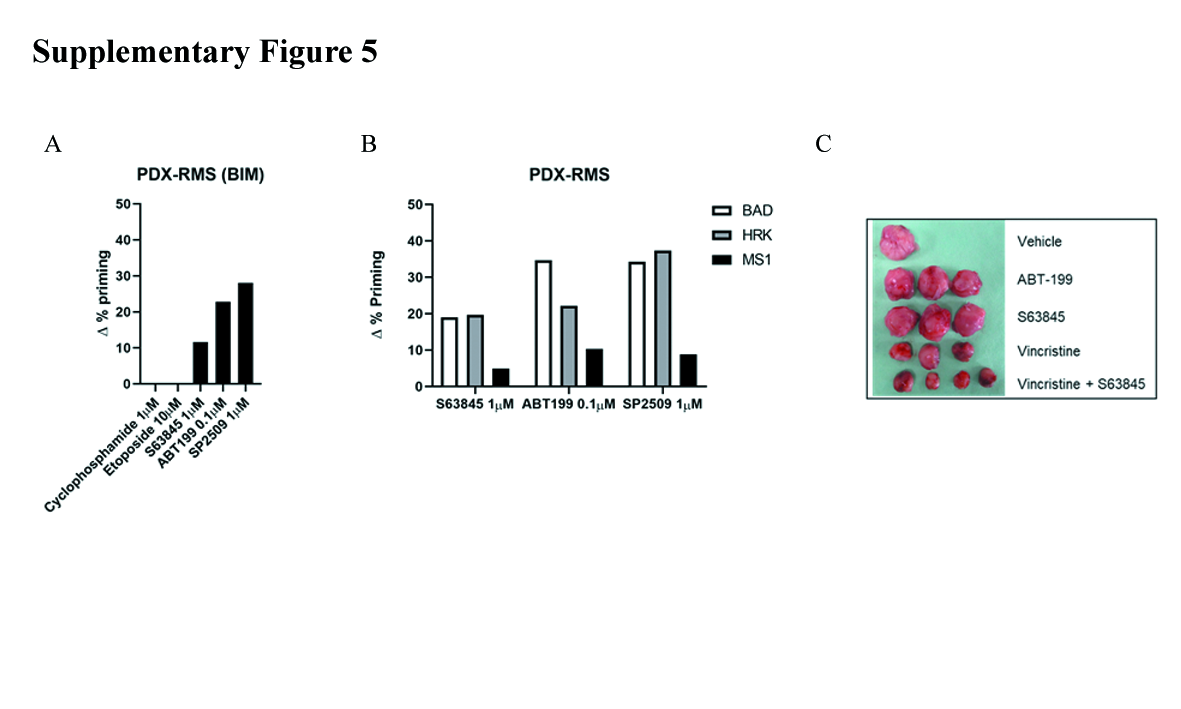

Supplement: Supplementary file 6 — Supplementary Figure 5 [file 41419_2020_2887_MOESM6_ESM.tif]
